# Enhancing Mesenchymal Stromal Cell Immunomodulation for Treating Conditions Influenced by the Immune System

**DOI:** 10.1155/2019/7219297

**Published:** 2019-08-05

**Authors:** Bella S. Guerrouahen, Heba Sidahmed, Asma Al Sulaiti, Moza Al Khulaifi, Chiara Cugno

**Affiliations:** Sidra Medicine, Member of Qatar Foundation, Doha, Qatar

## Abstract

Mesenchymal stromal cells (MSCs), formerly known as mesenchymal stem cells, are nonhematopoietic multipotent cells and are emerging worldwide as the most clinically used and promising source for allogeneic cell therapy. MSCs, initially obtained from bone marrow, can be derived from several other tissues, such as adipose tissue, placenta, and umbilical cord. Diversity in tissue sourcing and manufacturing procedures has significant effects on MSC products. However, in 2006, a minimal set of standard criteria has been issued by the International Society of Cellular Therapy for defining derived MSCs. These include adherence to plastic in conventional culture conditions, particular phenotype, and multilineage differentiation capacity *in vitro*. Moreover, MSCs have trophic capabilities, a high *in vitro* self-renewal ability, and immunomodulatory characteristics. Thus, immunosuppressive treatment with MSCs has been proposed as a potential therapeutic alternative for conditions in which the immune system cells influence outcomes, such as inflammatory and autoimmune diseases. The precise mechanism by which MSCs affect functions of most immune effector cells is not completely understood but involves direct contact with immune cells, soluble mediators, and local microenvironmental factors. Recently, it has been shown that their homeostatic resting state requires activation, which can be achieved *in vitro* with various cytokines, including interferon-*γ*. In the present review, we focus on the suppressive effect that MSCs exert on the immune system and highlight the significance of *in vitro* preconditioning and its use in preclinical studies. We discuss the clinical aspects of using MSCs as an immunomodulatory treatment. Finally, we comment on the risk of interfering with the immune system in regard to cancer formation and development.

## 1. Background

Mesenchymal stromal cells (MSCs) are nonhematopoietic cells which possess self-renewal, proliferative, and clonogenic potential and have the ability to commit to different cell types including adipocytes, chondrocytes, and osteocytes depending on the environmental conditions [1–3]. They can be easily isolated from human tissues and have exceptional biological properties for advanced therapies [4]. Traditionally derived from bone marrow (BM) [5], MSC populations may also be obtained from other various tissue sources, such as maternal decidua basalis of the placenta, adipose tissue (AT), foreskin, or neonatal birth-associated tissues (fetal part of the placenta and umbilical cord (UC)) [6, 7]. In 2006, the International Society for Cellular Therapy (ISCT) established the minimum criteria for designating MSCs derived from various origins: adherence to plastic in standard culture conditions; expression of different nonspecific surface molecules such as CD105/endoglin, CD90/Thy1, and CD73/5′-nucleotidase; lack of expression of CD34, CD45, CD14 or CD11b, CD79a or CD19, and HLA-DR (<2%); and trilineage differentiation potential due to the expression of several pluripotency genes. The weak expression of major histocompatibility complex (MHC) class I protects MSCs from natural killer (NK) cell-mediated killing; additionally, the lack of MHC class II expression confers to these cells the ability to evade immune recognition by CD4^+^ T cells. MSCs present minimal expression for HLA-DR (<2%) and do not express costimulatory proteins (CD80, CD86, and CD40), endothelial or hematopoietic surface molecule markers, such as CD31, CD45, CD34, CD14 or CD11b, and CD79a or CD19 [8]. New developments in characterization and marker profiling improve the methods of isolation, verification, and quality assessment of MSCs. In addition to hematopoietic support, tissue repair after injury, and use in regenerative medicine, the immunomodulatory properties of MSCs are attributes that represent the rationale for using MSCs as a novel therapy for many diseases, particularly disorders of the immune system [9–13]. Interestingly, the ISCT issued guidelines pertaining to MSC effector pathways such as immunomodulation, regeneration, and homing properties [14]. In 2002, for the first time, it was demonstrated that MSCs can modulate immunosuppression *in vitro* and *in vivo* [15]. For Caplan, the acronym MSC stands for “medicinal signaling cells,” indicating that the main attribute of MSC therapy is the secretion of bioactive molecules (extracellular vesicles (EVs), cytokines, growth factors, and chemokines) [16], and Caplan and Correa later proposed that the trophic and immunomodulatory properties of MSCs may function as site-regulated “drugstores” *in vivo* [17]. MSCs were also called the “guardians of inflammation” [18]. Those properties confer the clinical value of MSCs through the interaction with immune cells and the secretion of bioactive molecules leading to the suppression of lymphocyte proliferation, maturation of monocytes, and generation of regulatory T cells (Tregs) and M2 macrophages [19, 20]. In this review, we focus on the immunomodulatory effects of MSCs, the value of preconditioning, and its application in preclinical studies. We then comment on some clinical trials using MSCs and encountered hurdles. Finally, we discuss the risk of modulating the action of immune cells, which might theoretically favor the formation and development of cancer.

## 2. MSC-Mediated Immunomodulation of Immune Cells

MSCs were described as sensors of the inflammatory microenvironment in regard to their impact on the immune system [21]. Through cell-to-cell contact and regulatory molecule secretion which includes growth factors, chemokines, cytokines, and EVs, MSCs regulate both innate and adaptive immunity by affecting the activation, maturation, proliferation, differentiation, and effector functions of T and B lymphocytes (adaptive immune system), NK cells, neutrophils, and macrophages (innate immune system), as well as dendritic cells (DC), which link innate to adaptive immunity [22, 23].

### 2.1. T Lymphocytes

Activated T cells proliferate and release inflammatory cytokines and chemokines [24]. In the inflammatory environment, MSCs recruit local helper (Th) and effector T cells, via highly expressed chemokine (C-X-C motif) ligands CXCL9 and CXCL10, thus facilitating their immunomodulatory activity [25]. The intracellular enzymes indoleamine-2,3-dioxygenase (IDO) and inducible NO synthase (iNOS) produced by MSCs are some of the major mediators of T cell suppression, prompting their polarity shift from a proinflammatory Th1 state to an anti-inflammatory Th2 condition [26–28]. Galectin-1, abundantly expressed in and secreted by MSCs, also acts on T lymphocyte subpopulations and influences their cytokine production and release [29]. Interleukin- (IL-) 10, transforming growth factor- (TGF-) *β*, and the lipid mediator prostaglandin E2 (PGE2) secretion by MSCs inhibit Th17 cell differentiation and inhibit the production of IL-17, IL-22, interferon- (IFN-) *γ*, and tumor necrosis factor- (TNF-) *α* by mature Th17 cells [30–33]. In addition, TGF-*β* enhances T regulatory cell (Treg) function and differentiation, thus collectively modulating the Treg/Th17 balance [32]. Besides, the Notch 1 signaling pathway has been involved in MSC-mediated Treg differentiation [34], and the IL-10-dependent secretion of HLA-G5 further expands the Treg compartment [35].

### 2.2. B Lymphocytes

B cells are indispensable for humoral immunity and secrete antibodies when stimulated by antigens and inflammatory cytokines such as IL-10. Under quiescent conditions, MSCs trigger the differentiation into regulatory B cells (Bregs) [36]; while during inflammation, MSCs inhibit B cell proliferation, dampen the production of immunoglobulins (IgA, IgG, and IgM), and lose the capacity to induce Bregs [36–38]. While the potential of MSCs in B cell immunomodulation is not fully understood, it appears that inflammatory conditions are necessary for MSCs to exert their role through a combination of cell-cell contact (e.g., PD-L1 pathway) and soluble factors [39, 40].

### 2.3. NK Cells

Considered a subset of lymphocytes, NK cells are an important source of IFN-*γ* in addition to T cells [41]. MSCs are able to dampen the expansion of NK cells, effector functions, and cytotoxic production through the key mediators PGE2, IDO, and HLA-G5 [35, 42, 43].

### 2.4. Neutrophils

During inflammatory processes, neutrophils generate large concentrations of reactive oxygen intermediates and decrease the levels of antioxidants, which are regulators of the apoptotic cascade [44]. IL-6 produced by MSCs dampens respiratory bursts from neutrophils but does not affect phagocytic activity, matrix adhesion, and chemotaxis [45]. The suppression of their releasing destructive enzymes, such as peroxidases and proteases, rescues neutrophils from apoptosis [45, 46].

### 2.5. Macrophages

PGE2 secreted by MSCs influences the macrophage switch from an inflammatory M1 into an anti-inflammatory M2 state [47–49]. This M2 macrophage expresses high levels of CD206 and IL-10, reduces levels of TNF-*α* and IL-12, and shows higher phagocytic activity [50, 51]. In addition, the shift in macrophage polarization was observed *in vitro* and *in vivo* using EVs isolated from human AT-MSCs [52]. Morrison's group demonstrated this in an acute respiratory distress syndrome murine model using human-derived MSCs and postulated an EV-mediated mitochondrial transfer [53].

### 2.6. Dendritic Cells (DCs)

DCs, the most efficient antigen-presenting cells, prime naïve T cells to activate the adaptive immune cascade and interact with MSCs [54]. MSCs block the differentiation of monocytes towards DCs through a mechanism involving PGE2 [55] and prompt the differentiation of mature DCs into a regulatory subtype through cell-cell contact, involving Jagged-2 [56].

**Figure 1:**
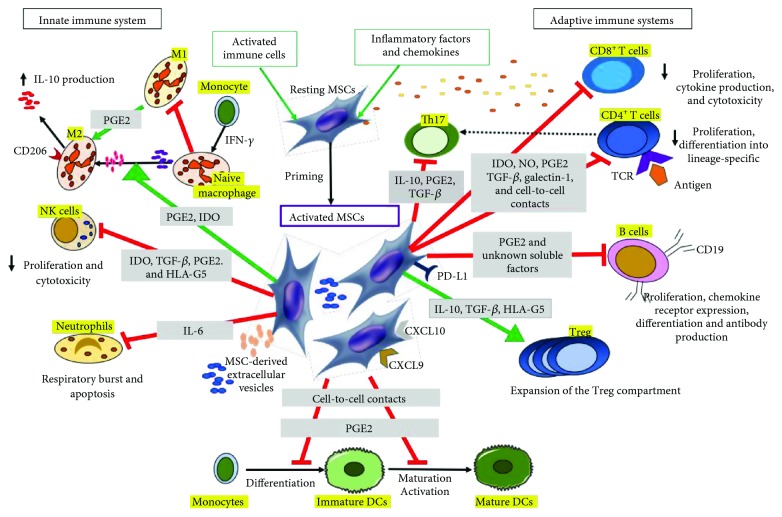
Mechanisms mediating immunomodulation. MSCs and their derived extracellular vesicles (EVs) exert their effect on innate (NK, neutrophils, monocytes, and macrophages) and adaptive (B and T cells) immune systems, as well as dendritic cells (DCs) through cell-to-cell interactions and several immunomodulatory factors. Activated T cells activate resting MSCs, which in turn facilitate the recruitment of helper and effector T cells via CXCL9 and CXCL10. Several immunomodulatory factors (TGF-*β*, PGE2, and HLA-G5) and membrane-bound molecules (PD-L1) suppress CD4^+^ and CD8^+^ T cell proliferation and induce the polarization of CD4^+^ T cells towards Th17 cells. NO and IDO released by MSCs act on the suppression of CD8^+^ T cell proliferation, cytokine production, and cytotoxicity. MSCs support the development of Treg populations via IL-10, TGF-*β*, and HLA-G5. In the context of B cells, MSCs inhibit activation, proliferation, chemokine receptor expression, and differentiation to antibody-secreting plasma cells. MSCs suppress naïve macrophage polarization to proinflammatory M1 macrophage and then favor anti-inflammatory M2 polarization. IL-6 secreted by MSCs suppresses neutrophil apoptosis and respiratory burst.

Figure 1 summarizes some of the mechanisms mediating immunomodulation.

## 3. Value of Preconditioning MSCs

### 3.1. Preconditioning MSCs to Enhance Immunomodulation

MSCs do not inherently display immunosuppressive properties at baseline. To replicate the inflammatory environment of a patient suffering from immune dysfunction, they require activation to adopt an immunosuppressive phenotype [57, 58]. In addition to the inflammatory status of the recipient, the efficacy of MSC-based therapies is influenced by differences in tissue origin, donor-to-donor heterogeneity, and dearth of standardized manufacturing practices [19, 21]. Ongoing research efforts are focused on “licensing” or “priming” MSCs to display a more homogeneous immunosuppressive phenotype. This concept refers to an *in vitro* exposure of MSCs to proinflammatory cytokines such as IFN-*γ*, TNF-*α*, IL-1*α*, or IL-1*β* [14]. Other preconditioning cytokines and stimuli such as hypoxia and pharmacological agents can also be used during *in vitro* culture to modulate the MSC secretory profile [59] and thus impact their properties [60]. Preconditioning strategies also extend to methods of triggering the expression of cytoprotective genes that aim at prolonging the longevity of MSCs introduced to an adverse inflammatory milieu and therefore extend the duration of the immunomodulatory effect exerted [61]. These stimuli appear to potentially “correct” such variation and therefore allow the use of more uniform therapeutic products with enhanced immunosuppressive potential, which may lead to higher clinical benefits in patients. Although strategies for improving MSC function are advancing at the bench, there are other factors to be considered before their implementation in the clinic. Nowadays, the assessment of functionally relevant markers reflecting the immunoregulatory properties of MSCs should become the basis for their clinical use as therapeutic cell-based products. Scientists at the U.S. Food and Drug Administration (FDA) designed an assay that identifies morphological changes associated with the immunosuppressive capacity after priming. By integrating the analysis of cellular changes with high-dimensional flow cytometry data and quantification of IFN-*γ*-augmented immunosuppression from multiple experimental conditions into a singular experiment, they were able to obtain a predictive measurement of the immunosuppressive capabilities of the cells [62].

### 3.2. Preclinical Studies Using Primed MSCs

Recent preclinical reports in the literature have demonstrated the significance of MSC priming with inflammatory cytokines for future clinical use. In addition to the aforementioned agents, others such as hyaluronan, polyinosinic acid, and polycytidylic acid have been used to prime MSCs for several forms of connective tissue repair in mice [63, 64]. These primed MSCs exhibit enhanced therapeutic properties with minimal or no significant adverse effects when compared to unprimed (naïve) counterparts [65, 66]. MSCs from multiple sources such as AT, BM, and Wharton's Jelly (WJ) primed with IFN-*γ* displayed gene expression profiles consistent with an immunosuppressive potential [67]. The immunomodulatory properties of MSCs derived from UC, AT, and periodontal ligaments presented comparable immunosuppressive capacities *in vitro*; however, UC-MSCs had shorter expansion time, predominantly utilized HLA-G as an immunosuppressive mechanism, and upon activation with IFN-*γ* did not express further HLA-DR, which would lower the risk of triggering an allogeneic immune response [68]. When IFN-*γ*-primed BM-MSCs isolated and cultured under good manufacturing practice (GMP) conditions were infused into murine models, no adverse effects related to primed BM-MSCs administration were found. Furthermore, the comparison of phenotypic profiles between primed and unprimed MSCs from the same donor demonstrated that the changes were due to IFN-*γ* priming rather than genetic variability [66]. In the context of graft versus host disease (GvHD), GvHD-mice injected with IFN-*γ*-primed MSCs had improved survival rates when compared to the group injected with naïve cells, and this was attributed to the activation of the IFN-*γ*-Janus kinase- (JAK-) signal transducer and activator of transcription 1 (STAT 1) pathway, which suppressed T cell proliferation [65].

## 4. Clinical Applications of MSCs in Diseases Mediated by Immune Cells

Culture-expanded MSCs are classified by both the FDA and European Medicines Agency (EMA) as more than minimally manipulated cellular and gene therapy (CGT) products [69]. The earliest therapeutic attempts at using autologous MSC infusion after ex vivo culture expansion showed an acceleration of the hematopoietic reconstitution after hematopoietic stem cell transplantation [70] and high-dose chemotherapy in breast cancer [71]. In both studies, no treatment-associated adverse effects were reported, thus these results laid the foundation for ex vivo cell expansion and administration. While the majority of MSC applications so far have relied on BM being the gold standard source, other adult and fetal tissues such as AT, UC, and WJ have gained popularity because of their comparable or even superior immunomodulatory profiles and their accessibility as medical waste products [72, 73]. For early phase human clinical trials, several factors including identity, viability, and sterility are established as release criteria [8]. However, for advanced-phase clinical trials, regulatory authorities additionally required the development of potency assays as part of the release criteria [74]. Additionally, the EMA has provided multiple guidelines to ensure quality, safety, and efficacy, including the “Guideline on Human Cell-Based Medicinal Products,” “Guideline on Strategies to Identify and Mitigate Risk for First-in-Human Clinical Trials with Investigational Medicinal Products,” and “Reflection Paper on Stem Cell-Based Medicinal Products,” among others [75].

### 4.1. Broad Range of Applications

Most of the clinical trials performed to date have showed the feasibility and safety of the approach with however conflicting results in terms of efficacy, partially explicable with methodological biases (i.e., small cohorts, lack of control groups, variability of source, and preparation and routes of administration). Also, the use of autologous *vs*. allogeneic MSC is still controversial with no univocal data on the immunological properties of MSCs derived from patients suffering from autoimmune disorders compared to healthy donors [76, 77]. We provide a brief overview of clinical trials performed or ongoing in the setting of immune-related disorders. However, a more comprehensive picture is beyond the scope of the current review.

Results of clinical trials in inflammatory bowel disease have been recently reviewed by Algeri et al. [76]. MSCs have been administered intravenously to control luminal inflammatory disease or locally in perianal fistulizing Crohn's disease (CD), in cases of refractory disease or acute flares not responsive to conventional methods of treatment such as steroids and immunosuppressive drugs. The two largest studies conducted on systemic administration of allogeneic MSCs have reached conflicting conclusions: Lazebnik et al. showed clinical response in all treated patients (39 Ulcerative Colitis and 11 CD, [78]), while Pfizer did not succeed to demonstrate any clinical benefit in 48 treated Ulcerative Colitis patients compared to 40 placebo [79].

More homogenous positive results have been obtained for the treatment of fistulizing CD where MSCs promote the healing of rectal mucosa, without any observable adverse events [80–82]. A phase III randomized, double blind, controlled trial with allogeneic, adipose-derived MSCs (Cx601) demonstrated a higher remission rate in 107 patients treated *vs*. 105 placebo [81]. Alofisel or Cx601 is going to be the first off-the-shelf MSC therapy to be approved by EMA for complex perianal fistulas in adult CD [83].

Since 2004, allogeneic MSCs have been used in the treatment of GvHD in several patients enrolled in a multitude of trials worldwide [10, 84]. Osiris sponsored a phase III trial of allogeneic BM-MSCs from random donors for the treatment of steroid-refractory GvHD (NCT00366145). Unfortunately, it was considered a failure due to a lack of positive outcomes [85]. This was due to inconsistencies in sourcing, isolation and manufacturing methods, passage numbers used, and fresh *vs*. thawed cells [86, 87]. Despite this, the Osiris-backed BM-MSC product has been approved in Canada, New Zealand, and Japan (on an insurance-dependent basis) for restricted use in children with GvHD [88]. Alternative sources have also been tested, and placenta-derived decidua stromal cells seem to hold promise of better response rates compared to BM-MSCs for severe acute GvHD [89].

Rheumatic disorders are also considered another potential area for MSC application. Since 2010, more than 300 patients with relapsing systemic lupus erythematosus (SLE) have been reported in the same center in Nanjing, China. However, the presence of multiple biases in the study design (i.e., lack of endpoint definition and of randomization) and in data analyses renders the study inconclusive in proving efficacy. Regardless, the use of pooled allogeneic MSCs derived from healthy donors was also shown to regulate and normalize lymphocyte counts and differentials in SLE patients [90].

Similarly, phase I/II uncontrolled clinical trials have been conducted in other inflammatory rheumatic diseases, such as systemic sclerosis, Sjögren syndrome, dermatomyositis/polymyositis, and rheumatoid arthritis with promising results, although bigger randomized prospective controlled studies are mostly warranted [91, 92]. Several ongoing clinical trials are exploring the efficacy and toxic effects of MSCs in patients with multiple sclerosis [93]; however, phase I/II studies have not brought significant positive results and further investigations are warranted [94, 95]. In a large nonrandomized comparative trial in 173 patients with active rheumatoid arthritis, the intravenous treatment with UC-MSCs succeeded in inducing a substantial remission of the disease as per the American College of Rheumatology improving standards [96]. Based on the fact that several studies in animal models of Type 1 Diabetes (T1D) have shown MSCs to ameliorate or reverse overt diabetes, also demonstrating their successful engraftment in the pancreatic islets [97, 98], Carlsson et al. performed a phase I clinical trial showing for the first time the opportunity to interfere with the progression of T1D by systemic infusion of MSCs. Autologous BM-MSCs were administered to adult patients recently diagnosed with T1D. Strikingly, during the first year postdiagnosis, no adverse events were disclosed and a conserved or improved C-peptide response to a mixed-meal tolerance test in the patient cohort was demonstrated [99].

Table 1 summarizes other clinical trials of MSCs on diseases mediated by the immune system not previously discussed (http://www.clinicaltrials.gov, [100–106]).

### 4.2. Current Challenges in Clinical Use

#### 4.2.1. Fate of the Infused MSCs

A factor that influences the future of MSC application in the clinic is that the exact fate of the cells postinfusion is yet to be completely elucidated. There are multiple reports in both human and animal models that point to sequestration of the cells in the lungs following systemic administration and their complete disappearance within 7 days of treatment [9, 107, 108]. Another study showed that allogeneic donor MSC DNA was found engrafted into the recipient's digestive tract *via* chromosomal fluorescence studies [10]. This is in support of the theory that MSCs are capable of escaping sequestration and migrating to sites of inflammation, homing to released cytokines and other inflammatory molecules. If this is the case, this will facilitate the administration of MSCs to patients with multisystemic or disseminated involvement, e.g., SLE and rheumatoid arthritis, with gross effects including treating inflammation, regulating lymphocyte function, and stimulating tissue repair, including regeneration of cartilage [109]. Other theories suggest that MSCs prior to apoptosis release EVs that are capable of migrating to inflamed tissues and exerting the same anti-inflammatory effects of viable MSCs. This alternative approach highlights the potential of cell-free MSC-based therapy [52, 107].

#### 4.2.2. Practical Decisions Impacting MSC-Based Therapy Outcome

Other dilemmas impacting the widespread clinical use of MSCs that researchers have yet to reach a consensus for are which tissue source yields the most effective product, combined with the significant impact of donor variability and continued passaging on cell growth, protein production, and EV release [85, 110]. Furthermore, there is a lack of standardized disease-specific procedures and clinical trial regulations regarding the magnitude (average of 1-2 million cells/kg body weight) and frequency of dose administration, the use of allogeneic *vs*. autologous MSCs, systemic *vs*. local administration, and primed *vs*. naïve cells, and the use of freshly cultured *vs*. frozen and thawed cells [76]. Functional differences were observed between *in vivo* and *in vitro* contexts and between species (murine *vs*. human) in terms of susceptibility to undergoing oncogenic transformation during expansion, and effector molecules used in T cell suppression mechanisms have to be taken into account [21]. This is highlighted by the reported discrepancies between what is described in *in vitro* and animal models *vs*. what is reported in the literature of later-phase clinical trials and by the publishing bias (few or no reports on negative outcomes and/or failed trials) [92]. Interestingly, the lack of consistent benefit seen in late phase human clinical trials may also be explained by the fact that the injected cell products were “naïve or resting” MSCs; therefore, the immunosuppressive potential of the cells is entirely depending on an individual patient's microenvironment and immune status [19, 21, 111]. These variables collectively hinder the production of reliable “off-the-shelf” cell therapy products that produce sustainable and consistent results among patients.

## 5. Risk of Modulating the Action of Immune Cells and the Dilemma of Cancer Formation and Development

One of the main concerns in MSC-based therapy is that tumorigenicity could result from MSC malignant transformation during *in vitro* culture expansion or following infusion, or the immunosuppressive effects exerted by MSCs could allow tumor formation and development of already existing malignant cells in the host/recipient [112]. Similarly to murine MSCs readily undergoing spontaneous transformation *in vitro* [113], Rosland et al. demonstrated spontaneous malignant transformation of BM-derived human MSCs after *in vitro* cultures leading to an aggressively metastatic disease in immunodeficient mice [114]. However, the impaired immunological status of the recipient was likely more prone to initiate or develop cancer [115]. In humans, MSCs are minimally susceptible to oncogenic transformation *in vivo*, and long-term culture either does not affect MSC morphology or cause chromosomal alterations [116]. Furthermore, continued passaging leads to loss of already existing aneuploidy, or any resulting aneuploidy leads to senescence, negating the risk of cancer formation [117]. The Committee for Advanced Therapies and the Cell Product Working Party organized a meeting to discuss the risk of tumor formation following MSC-based therapies, with a focus on regulatory and scientific aspects. When discussing the influence of the manufacturing process on inducing cytogenetic abnormalities, it was highlighted that culture duration and conditions present critical risk factors for producing chromosomal aberrations. The committee also suggested that long-term expansion could mostly cause chromosomal aberrations rather than donor-derived factors [112]. However, in a study by Tarte et al., aneuploidy without risk of transformation occurring in a long-term culture of clinical grade MSCs was most likely donor dependent (3 out of 5 aberrations were derived from the same donor) [118]. Thus, donor screening and monitoring of the long-term expansion and integrity of the cells are a requirement [119].

MSCs exhibit a tropism for the tumor microenvironment niche [120], and selective homing into inflammatory tumor sites has been established in various types of cancer [121]. Even if MSCs have intrinsic antitumor properties, they can potentially alter their phenotype towards a protumorigenic role including proangiogenic and immunosuppressive capabilities. Thus, the presence of MSCs within the cancerous stroma has been a matter of contradictory reports [122]. There is no official statement on the potential of tumorigenicity in MSC-based therapies, and no observation of tumor formation of MSC origin in patients given cellular therapy. Despite these facts, one cannot rule out the possibility of MSC-derived tumors developing *in vivo*. Interestingly, there are reports of spontaneous MSC transformation resulting from MSC culture cross-contamination with malignant cells emphasizing the importance of maintaining good manufactory practice conditions in the production of cell therapy products [123, 124]. While MSC therapy has been qualified as safe by both FDA and EMA, the potential long-term risks still have to be considered.

## 6. Conclusion

In the last 10 years, MSCs have been a promising treatment for a plethora of immune-related conditions, through the regulation of inflammation and the support of tissue homeostasis. Despite having been unanimously deemed safe, clinical trials report conflicting data in terms of efficacy in several clinical settings. Inconsistencies can be ascribed to limitations in the design of clinical trials and translation of successful preclinical models, discrepancies in the source, preparation and handling of the MSC product, route of administration, and type of donor (autologous *vs*. allogeneic).

Moreover, the lack of *in vitro* biomarkers correlating with the *in vivo* activity of MSCs has so far hindered the progress towards uniformly potent cell products. MSC priming or licensing, before administration, might offer the possibility to enhance their effectiveness *in vivo*, limiting the variability inherent to the inflammatory status of the patients.

## Figures and Tables

**Table 1:** Clinical trials of MSCs on diseases mediated by the immune system.

Trial no.	Phase	Commencement year	Targeted disease	Status	Patient enrollment (*n*)	Country
NCT00447460	I/II	2007	Graft vs. host disease (GvHD)	Completed [100]	15	Spain
NCT01522716	I	2011		Unknown (NRP)	11	Sweden
NCT01764100	I	2013		Completed [101]	40	Italy
NCT02032446	I/II			Recruiting	47 (estimated)	
NCT02291770	III	2014		Unknown (NRP)	130 (estimated)	China
NCT02055625	I/II			Suspended (NRP)	11	Sweden
NCT02359929	I	2015		Recruiting	24 (estimated)	USA
NCT01741857	I/II		Systemic lupus erythematosus (SLE)	Completed [102]	40	China
NCT03171194	I			Active, not recruiting	6 (estimated)	USA
NCT03673748	II	2019	SLE/lupus nephritis	Not yet recruiting	36 (estimated)	Spain
NCT00781872	I/II	2006	Multiple sclerosis (MS)	Completed [103]	20	Israel
NCT00395200	I/II	2008		Completed [104, 105]	10	UK
NCT01730547	I/II	2013		Unknown	15 (estimated)	Sweden
NCT02495766	I/II	2015		Unknown	8 (estimated)	Spain
NCT03799718	II	2019		Not yet recruiting	20 (estimated)	USA
NCT02893306	II	2012	Type 1 diabetes mellitus (T1DM)	Unknown (NRP)	10	Chile
NCT02940418	I	2017		Recruiting	20 (estimated)	Jordan
NCT03406585	I/II			Recruiting	24 (estimated)	Sweden
NCT02249676	II	2013	Devic syndrome/neuromyelitis optica	Completed	15	China
NCT01659762	I	2012	Crohn's disease	Completed [106]	16	USA

NRP: no results posted.
